# Artonin E and Structural Analogs from *Artocarpus* Species Abrogates Estrogen Receptor Signaling in Breast Cancer

**DOI:** 10.3390/molecules21070839

**Published:** 2016-06-29

**Authors:** Imaobong Etti, Rasedee Abdullah, Najihah Mohd Hashim, Arifah Kadir, Ahmad Bustamam Abdul, Christopher Etti, Ibrahim Malami, Peter Waziri, Chee Wun How

**Affiliations:** 1 Pharmacology and Toxicology, Faculty of Veterinary Medicine, Universiti Putra Malaysia, Serdang 43400, Malaysia; 2 Department of Pharmacology and Toxicology, University of Uyo, Uyo 520271, Nigeria; 3 Department of Veterinary Pathology and Microbiology, Faculty of Veterinary Medicine, University Putra Malaysia, Serdang 43400, Malaysia; 4 Department of Pharmacy, Faculty of Medicine, University of Malaya, Kuala Lumpur 50603, Malaysia; najihahmh@um.edu.my; 5 Department of Veterinary Preclinical Science, Faculty of Veterinary Medicine, Universiti Putra Malaysia, Serdang 43400, Malaysia; arifah@vet.upm.edu.my; 6 MAKNA-Cancer Research Laboratory, Institute of Bioscience, Universiti Putra Malaysia, Serdang 43400, Malaysia; zer2crystals@gmail.com (A.B.A.); keepibinformed@yahoo.co.uk (I.M.); petermwaziri@gmail.com (P.W.); 7 Department of Agricultural and Food Engineering, University of Uyo, Uyo 520271, Nigeria; christopheretti@uniuyo.edu.ng; 8 Laboratory of Vaccine and Immunotherapeutics, Institute of Bioscience, University Putra Malaysia, Serdang 43400, Malaysia; cwhow2000@yahoo.com

**Keywords:** in silico, Artonin E, molecular docking, human estrogen receptor, *Artocarpus*

## Abstract

The increasing rate of mortality ensued from breast cancer has encouraged research into safer and efficient therapy. The human Estrogen receptor α has been implicated in the majority of reported breast cancer cases. Molecular docking employing Glide, Schrodinger suite 2015, was used to study the binding affinities of small molecules from the *Artocarpus* species after their drug-like properties were ascertained. The structure of the ligand-binding domain of human Estrogen receptor α was retrieved from Protein Data Bank while the structures of compounds were collected from PubChem database. The binding interactions of the studied compounds were reported as well as their glide scores. The best glide scored ligand, was Artonin E with a score of −12.72 Kcal when compared to other studied phytomolecules and it evoked growth inhibition of an estrogen receptor positive breast cancer cells in submicromolar concentration (3.8–6.9 µM) in comparison to a reference standard Tamoxifen (18.9–24.1 µM) within the tested time point (24–72 h). The studied ligands, which had good interactions with the target receptor, were also drug-like when compared with 95% of orally available drugs with the exception of Artoelastin, whose predicted physicochemical properties rendered it less drug-like. The in silico physicochemical properties, docking interactions and growth inhibition of the best glide scorer are indications of the anti-breast cancer relevance of the studied molecules.

## 1. Introduction

The human estrogen receptors belong to the nuclear family of receptors and play a critical role in reproduction and normal physiology [1]. They are reported to have two subtypes, viz., the human estrogen receptor α (hERα) and human estrogen receptor β (hERβ) [2]. In spite of the importance of these receptors in cellular behaviors, their abilities to induce cell proliferation is central to their roles in breast cancer, a disease with untold burden to the world. Among the two subtypes, the hERα status has been reported as the most important predictor of breast cancer prognosis [3]. At the time of breast cancer diagnosis, about 70% of all human breast cancers express hERα [4]. Treatment strategies for estrogen receptor positive cancers involve blocking the action of the receptor; either by inhibiting estrogen production using aromatase inhibitors, or by interfering with the binding of estrogen to its receptor using selective estrogen receptor modulators (SERMs) such as Tamoxifen [5]. Unfortunately, resistance is very common with conventional therapeutic strategies, decreasing the survival rate [6]. The low rate of survival and ensued resistance to current therapy prompted the exploration of small molecules, especially from plant sources, which can inhibit the hERα, being the mostly expressed subtype of breast cancer and hence curb its signaling.

The Moraceae plants have been widely investigated for their rich phytochemicals and one of the main genera in this family is *Artocarpus*, which is distributed in tropical areas of the globe. *Artocarpus* species are evergreen Asiatic trees with extruded white latex and fleshy fruit containing lots of seeds [7]. The fruits, roots, bud and leaves of *Artocarpus* have been widely used as traditional medicine for the treatment of malarial fever, liver cirrhosis, hypertension and diabetes [8,9]. It is exceptionally rich in phenolic secondary metabolites such as flavonoids, chalcones, xanthones and arylbenzofurans [10,11]. Some of these constituents have been reported to possess anti-inflammatory, anti-proliferative [12,13], antimicrobial [12] anti-tubercular [10] and antioxidant properties [14]. However, small molecules from this *Artocarpus* have not been investigated for their binding affinities to the human estrogen receptor and their drug-likeness has not been reported. This study examined the binding affinities of the following molecules from the *Artocarpus* genus: Artonin E, Artobiloxanthone, Cycloartocarpesin, Artoelastin, Artonin Y, Artonin U, Artonin L, Artonin T and Artonin S.

Computational modeling is an essential component in modern drug discovery and has proven very useful in the screening and selection of potent inhibitors [15]. It has offered an efficient tool in predicting the possible interactions between the studied ligands and the active site of the target receptors [16], which enhances structure based drug design. Docking studies partly replace the laborious and time consuming in vitro screening and has been extensively employed by pharmaceutical companies in screening for lead compounds during drug discovery [16]. In this context, Malami et al. [17] have recently demonstrated the applicability of molecular docking studies in discovering potential uridine cytdine kinase 2 inhibitors from the rhizomes of *Alpinia mutica*.

The objective of this study was to utilize molecular docking to explore possible small molecule inhibitors from *Artocarpus*, examine their binding efficiencies to the ligand-binding domain of the hERα and preliminarily test for the in vitro anti-breast cancer relevance of the best glide scorer among the investigated phytomolecules in halting undue proliferation of an estrogen receptor positive breast cancer cell line. This study will help in the development of new estrogen receptor modulators to prolong the rate of breast cancer survival.

## 2. Results

### 2.1. Prediction of Drug-Likeness

Poor physicochemical properties of drugs have often led to the exit of promising drug molecules from clinical trial despite the huge cost and labour involved in the preclinical testings. Most of the reasons for this observation has been attributed to poor physicochemical properties of such compounds [18,19]. Today, with the help of computational techniques, accurate physicochemical properties can be predicted prior to expensive experimental procedures [20]. This computational analysis of drug-likeness is very crucial during drug discovery, as it predicts descriptors of the drug molecule which can be enhanced before taking such compound for further pharmacological analysis [19]. It is obvious that no molecule intended for oral route will produce any potential pharmacological effect except when absorbed via the biological membrane. This process is, however, influenced by certain characteristics of such molecule, which were examined in this study. To evaluate the physicochemical properties of the ligands, certain descriptors, reported to correlate with good oral bioavailabilty [21] were taken into consideration, including the Lipinski’s rule of five, which predicts that poor oral absorption or permeation is more likely to occur when there are more than “5 *H*-bond donors, 10 *H*-bond acceptors, molecular weight of more than 500 and the calculated Log P (CLogP) is greater than 5” [18]. The Qikprop module of Schrodinger [22], was used to predict the drug-likeness of the studied compounds. Qikprop compares these predicted descriptors with those of 95% of known orally available drugs (Table 1). The Properties evaluated include: aqueous solubility, molecular weight, octanol/water partition coefficient, estimated number of hydrogen bonds that would be donated and accepted, total solvent accessible surface area, predicted apparent Caco-2 cell permeability in nm/s (a model for gut-blood barrier), predicted brain/blood partition coefficient, number of likely metabolic reactions and predicted human oral absorption on 0% to 100% scale (see Table 1). Comparing the results obtained (Table 1) with those of 95% orally available drugs with respect to the above stated Lipinski rule of five, all the studied ligands had excellent molecular weight, donor and acceptor hydrogen bonds and a better predicted octanol/water partition coefficient (QPlogPo/w), except for Artelastin, Tamoxifen and the native ligand, whose QPlogPo/w, and molecular weight resulted in one violation of the Lipinski’s rule of five. This one violation showed by these compounds is, however, accommodated in qikprop. The ligands also had perfect predicted aqueous solubilities (QPlogS) and human oral availabilities with the exception of the native ligand whose oral availability was moderate (<80%). A previous study by Veber et al. [21], showed that these predicted descriptors correlate well with in vivo bioavailabilty and are critical in developing oral dosage. From the predicted number of metabolic reactions, which indicated the possible number of biotransformation of the compound, Artelastin, unlike other ligands also exceeded the predicted limit. The pemeability of the ligands accessed with in silico Caco-2 model, showed that all the studied compounds were compliant. The Caco-2 model is the most popular and extensively characterized cell-based model employed in pharmaceutical industries and academic research fields in predicting drug permeability [23,24]. The predicted total solvent accessible surface area (SASA) and brain/blood partition coefficient were also within the recommended range for orally available drugs, except the native ligand, which was >1000 (Table 1). Amongst the studied phytomolecules, Artelastin was less drug-like when compared to the rest. The prediction was done with respect to the oral route of drug administration, which is still the most preferred route for new chemical entities (NCEs), in spite the advances in drug delivery methods. This uniqueness is owed to its convenience, low cost and high patient’s compliance. All descriptors analyzed and reported are vital for an orally administered drug to achieve a therapeutic concentration [18].

### 2.2. Docking Assessment

#### 2.2.1. Structure of the Human Estrogen Receptor α, 2IOG

**Figure 1:**
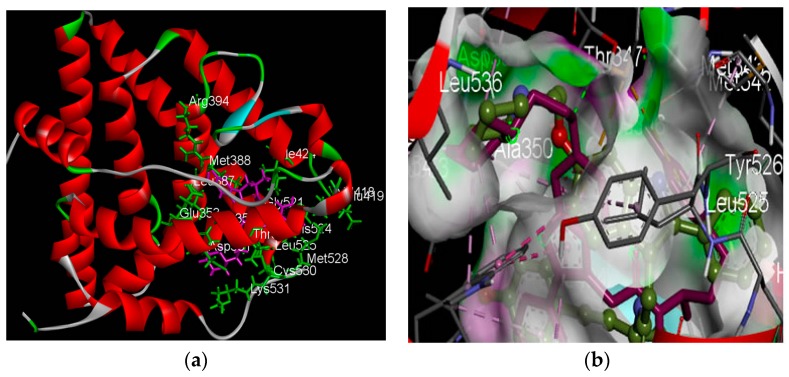
X-ray Structure of PDB ID 2IOG: (**a**) Target receptor, 2IOG with co-crystallized native ligand shown in purple sticks and amino acid residues shown as green sticks with their three-letter code and name; and (**b**) redooked native ligand superimposed with the crystallized native ligand within the binding pocket of 2IOG showing hydrogen bonding in white surface cartoon.

The three-dimensional structure of the hERα was retrieved from the Protein Data Bank with PDB ID: 2IOG determined by X-Ray crystallography at a resolution of 1.60 (Å) and visualized in Discovery studio. The complete X-ray structure of the protein (Figure 1a) is depicted with amino acid residues (viewed within 5 angstrong) shown as green sticks and labeled with their three-letter code. The native ligand, compound 11F, being a co-crystal structure of the target protein, is depicted as purple sticks (see Figure 1a). This particular PDB receptor was chosen based on its crystallographic resolution and species of interest.

#### 2.2.2. Identification of Estrogen Receptor-Binding Pockets and Validation of Docking Protocol

The human Estrogen receptor catalytic site predictions were carried out using the Cast p program [25]. The server measured analytically the area and volume of each pocket. The best ligand binding site was observed to be at pocket no. 36 of volume 1178.9Å3 and area of 901.1Å2, and consisted of 36 residues ID: Met343, Leu346, Thr347, Leu349, Ala350, Asp351, Glu353, Leu354, Trp383, Leu384, Leu387, Met388, Leu391, Arg394, Phe404, Val418, Glu419, Gly420, Met421, Ileu424, Phe425, Leu428, Gly521, His524, Leu525, Tyr526, Met528, Lys529, Cys530, Lys531, Asn532, Val533, Val534, Pro535, Leu536, and Leu539. Some of these amino acids viewed within 5 angstrong are depicted in Figure 1a after visualizing with discovery studio. This identification is similar to the findings of Suganya et al. [26] and reveals possible residues around the active site with which a potential ligand(s) can bind [27].

**Figure 2:**
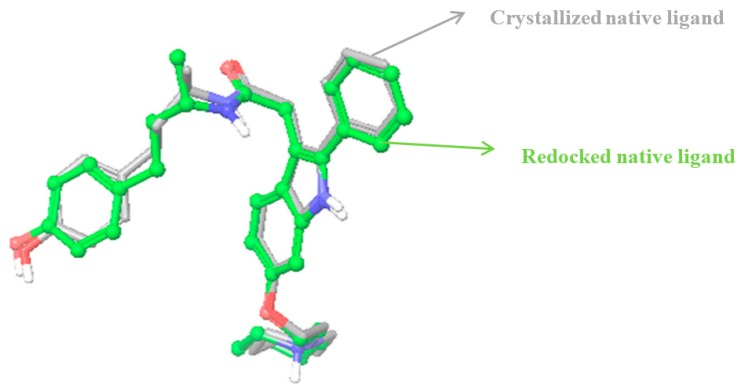
Docking control. Top ranked ligand pose for the native ligand, compound 11F 3-(4-hydroxyphenyl)-1-methylpropyl]-2-[2-phenyl-6-(2-piperidin-1-ylethoxy)-1h-indol-3-yl]acetamide-LBD hERα superimposed with co-crystallized native ligand.

To validate the docking protocol, the root mean square deviation between the co-crystallized native ligand and the redocked native ligand should be within 2 angstrong as can be visibly appreciated when the redocked and co-crystal structures are superimposed and the deviation calculated. When comparing the co-crystallized structure of compound 11F, the native ligand to the estrogen receptor, 2IOG, with our redocked compound 11F, the root mean square deviation obtained was 0.7864. Their superimposition was also correctly reproduced (Figure 2) within the binding domain of the target receptor (see Figure 1b). For the purpose of clarity, the receptor was excluded and only the superimposed structures shown in Figure 2.

#### 2.2.3. Docking Analysis

**Figure 3:**
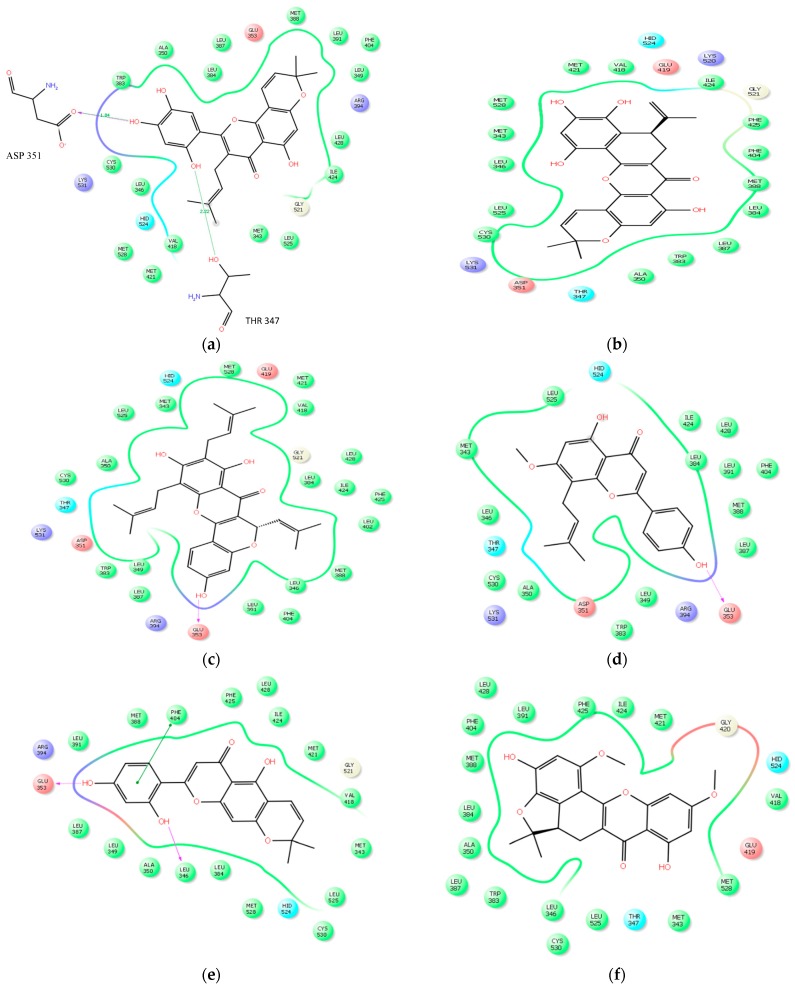

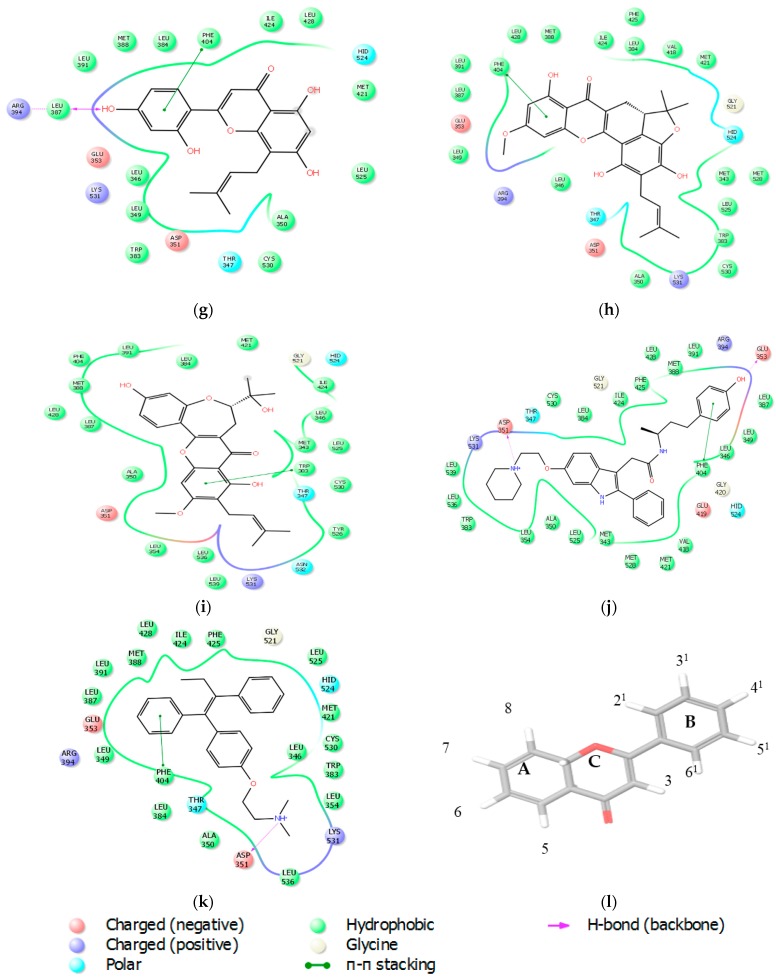
Molecular interactions of studied ligands with crucial amino acids at the ligand-binding domain of hERα: (**a**) Artonin E; (**b**) Artobiloxanthone; (**c**) Artelastin; (**d**) Artonin U; (**e**) Cycloartocarpesin; (**f**) Artonin L; (**g**) Artonin Y; (**h**) Artonin T; (**i**) Artonin S; (**j**) Native ligand, 11F; (**k**) Tamoxifen; and (**l**) A flavone skeleton.

The studied molecules were docked alongside with the native ligand and a reference standard, Tamoxifen, which served as controls. The results of the electrostatic interaction between the studied molecules and the target receptor are as depicted in Figure 3a–k. An estimation of the binding affinity, depicted as the glide or docking score was used to access the binding affinities of the studied ligands to the target receptor (see Table 2), increased in electronegativity is a function of the binding affinity [28,29]. Glide has been observed to be more accurate than other docking tools such as GOLD and Surflex methods [28]. Kosh et al. [30], estimated during their in silico studies on phthalates that a Glide score greater than −7 kcal/mol is considered as promising, and that it can go as high as −13 or even more. This study revealed glide scores between −16.81 and −9.10, indicating good affinities to the target receptor (Table 2). All the studied ligands had good Glide scores with the most outstanding score being Artonin E (−12.72) as compared to other molecules from *Artocarpus* species (see Table 2). The best three glide scorers also showed the best prime ∆G binding energy as depicted in Table 3. The roles of certain crucial amino acids in the ligand-binding domain of the human estrogen receptor α, was also established.

Major non-covalent interactions between the studied ligands and the ligand-binding domain of the hERα was investigated using key amino acids within the receptor. These amino acids have been repeatedly implicated during ligand interaction with the hERα [31] and also play important role in the inhibition of the ligand-binding domain of hERα [26,29,31]. This non-covalent interactions: Van der Waals, columbic interaction, π-π interaction and hydrogen interaction are shown in Table 2 and Figure 3a–l.

Structuarally, all the studied molecules each contain the basic flavone skeleton, i.e., comprising of two benzene rings (A and B as shown in Figure 3l) linked by a three carbon chain that form a closed pyran ring (C). Some of the compounds are prenylated (see Figure 3a,c,d,g–i), while others are not prenylated. Alternatively, the compounds can as well be seen in two forms: those in which the carbon chain forming the C ring is not occupied by any ring structure (Figure 3a,d,e,g or those in which the C ring linking carbon chain is occupied by other ring structures (see Figure 3b,c,f,h,i). From the results as seen in Figure 3a, the 2^1^–OH and 4^1^–OH groups of Artonin E established 1H bond each with negatively charged Aspartate 351, believed to be necessary for antagonism [32], and polar Threonine 347 at distances of 1.84 Ǻ and 2.88 Ǻ respectively (see Figure 3a). The 4^1^–OH groups of Artoelastin, and Artonin U each formed 1H bond with negatively charged glutamate 353 (Figure 3c,d). In Figure 3e, Cycloartocarpesin was observed to form 2H bonds, at 2^1^–OH group with negatively charged Glu 353 and at 4^1^–OH group with hydrophobic Leu 346. The 4^1^–OH group of Artonin Y (Figure 3g) formed 2H bonds with hydrophobic Leu 387 and positively charged Arg 394. Worthy of note is that the hydrogen bond distances, showed in Figure 3a–k, excluded distances beyond 3 Ǻ. However, distances within 4 Ǻ have been reported in Table 4. It is also evident that the benzene rings of cycloartocarpesin (Figure 3e), Artonin Y (Figure 3g), Tamoxifen (Figure 3k) and Artonin T (Figure 3h) each, formed a π−π interaction with Phe 404 (see Figure 3e,g,h) respectively, while Artonin S (Figure 3i) formed a π−π interaction with Phe 383. Interestingly, all the hydrogen bond interactions appeared to be formed predominantly in the B ring of the flavone skeleton of the ligands (Figure 3a–i). This may be attributed to the vicinal diol groups attached to this ring which enhances their binding to the target receptor. Worthy of note is the observation that the prenylated group together with the 4^1^, 5^1^ vicinal diol of Artonin E appeared to have enhanced the ligand’s binding affinity to the target receptor as shown in its firm hydrogen bond interaction involving the 4^1^, 5^1^ vicinal diols which may be attributed to its high glide score when compared to the other phytomolecules. These vicinal diol group were earlier reported by Reddy et al. [33] to improve the compound’s inhibitory activity towards arachidonate acid.

The clustering of active amino acids around the studied ligands strengthened the ligands anchorage to the target receptor (see Figure 3a–k), the green solid line in each Figure shows hydrophobic interactions with amino acids in the ligand-binding domain of the receptor and the purple arrow indicate the strongest hydrogen bonding interaction of <3 angstrong.

Resistance to current anti-estrogens like Tamoxifen and the recurring cases of breast cancer [34] prompted the attention to search for potential estrogen receptor targeting small molecules from nature. Glide of the Schrodinger suite 2015, was employed to study the binding affinities of some small molecules isolated from the *Artocarpus* species with the view of finding a potent inhibitor for ligand-binding domain of the human estrogen receptor α. Evidently, the results revealed for the first time, the relative potential of the studied ligands in abrogating estrogen signaling, a unique property in targeting estrogen positive breast cancers.

### 2.3. Prime Energy Analysis

The Prime molecular mechanics generalized born surface area (MM-GBSA) binding energy was calculated for the studied ligands with the program prime, of the Shrodinger suite [35]. The output properties calculated include: Prime Coulomb energy of the complex ($\Delta G_{bind}$ coulomb), Prime Van der Waals energy of the complex ($\Delta G_{bind}$ vdW), Prime energy of the complex (Prime MMGBSA complex energy), Prime MMGBSA Ligand Energy, Prime MMGBSA Receptor Energy, Prime MMGBSA $\Delta G_{bind}$ (kcal/mol) and Prime hydrogen bond ($\Delta G_{bind}$ Hbond). The results from the prime energy calculations are as shown in Table 3 with the free energy of binding, $\Delta G_{bind}$ (kcal/mol) calculated as described in Section 3.4 (Equation (1)).

### 2.4. In Vitro Growth Inhibition Assay

**Figure 4:**
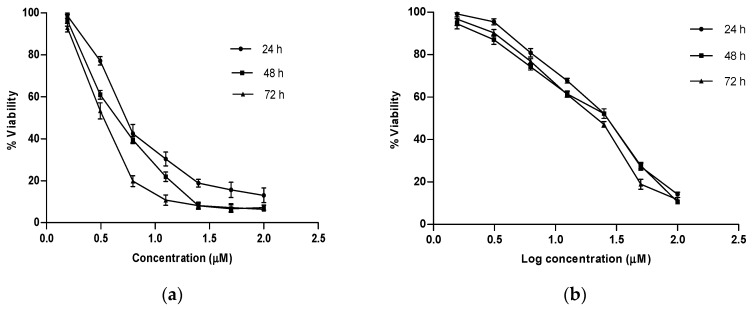
Dose–response curves of time-interval effect for: (**a**) Artonin E and (**b**)Tamoxifen on MCF 7.

To test the prediction of anticancer relevance of the docking studies, we investigated the growth inhibitory potential of the best glide scorer among the studied phytochemicals, Artonin E and a reference standard, Tamoxifen, in estrogen positive human breast cancer cells, MCF 7 using varying concentrations of the compounds at three different time points. Dose–response curves were plotted for each of the compounds (see Figure 4a,b) and the concentration of tested agents which evoked a 50% growth inhibition of the breast cancer cells were determined, as shown in Table 5 along with their 95% confidence intervals. It was observed that the reference standard, which showed a better docking score of 13.93 kcal, was unable to evoke a stronger in vitro inhibition on the estrogen receptor positive breast cancer cells as compared to Artonin E, whose score was 12.72 kcal. This observation, though consistent with other reported studies [36], was suspected to be due to phenol red, a component of the growth media with which the cells were raised before treatment [37]. The in vitro growth inhibition of the native ligand with a docking score of 16.81 Kcal as previously reported by the crystallographers was 42.7 nM [32] in comparison to Artonin E (docking score of 12.72 kcal and in vitro inhibition of 3.8 µM at 72 h). Generally, the growth inhibition of these compounds was observed to improve upon increasing concentration and time exposure (data available in Supplementary Materials). Comparing the time interval effect, the means of the different growth parameters were statistically significant (*p* < 0.05) when compared to the values of the 24 h time point as analyzed using Analysis of Variance in GraphPad prism 5.0 (GraphPad Software Inc., La Jolla, CA, USA).

## 3. Materials and Methods

### 3.1. Preparation of Ligands

The structures of the ligands were downloaded from the PubChem database and their 3D structures were prepared with Maestro, using ligprep [38], a utility of Schrodinger software suite that combines tools for generating accurate and high quality 3D molecular model from 1D (Smiles) and 2D (SDF) representations. The ligprep applied energy minimization with optimized potentials for liquid simulations-2005 (OPLS_2005) as the applied force field and filtered the ligands before they were used for further computational studies. The output structures were finally written to a file in maestro format.

### 3.2. Determination of ADMET Properties of the Compounds

Most promising drug candidates often fail during clinical trials owing to poor drug-like characteristics. To nominate potential drug candidates among the studied compounds, certain properties which relate to absorption, distribution, metabolism and excretion were investigated [39]. The QikProp module of Schrodinger Suite was used to predict the following parameters of the studied molecules: absorption, distribution, metabolism, and excretion. In addition to predicting molecular properties, QikProp also provides ranges for comparing a particular molecule’s properties with those of 95% of known orally available drugs. QikProp predicts physically significant descriptors and pharmaceutically relevant properties of organic molecules, either individually or in batches.

In the present study, QikProp was run in a normal processing mode with default options (Qikprop 4.6). The selected properties that are known to influence metabolism, cell permeation and bioavailability are presented in Table 1. These properties were thereafter compared with 95% of known oral drugs and also tested with the Lipinski’s rule of five to be considered as drug-like.

### 3.3. Molecular Docking Studies

#### 3.3.1. Identification of Binding Pockets and Validation of Docking Protocol

Usually, binding sites and active sites of proteins are often associated with structural pockets and cavities. The castP server was employed for this identification. This program uses the weighted Delaunay triangulation and the alpha complex for shape measurements [40]. It provides identification and measurements of surface accessible pockets as well as interior inaccessible cavities, for proteins and other molecules. It also measures the number of mouth openings, area of the openings, and circumference of mouth lips, in both SA and MS surfaces for each pocket [25] as reported in the results above (see Section 2.2.2).

The predictive ability of the docking protocol was validated by redocking the native ligand, compound 11F to hERα (Protein Data Bank (PDB) ID code 2IOG). To prove the appropriateness of the utilized docking protocol, the native ligand, which was co-crystallized to the target receptor as available in the protein data bank was redocked back to its receptor and such orientation compared by superimposing the co-crystallized ligand to the redocked native ligand and computing the root mean square deviation between them. The root mean square deviation between them should be within 2 angstrong before such docking protocol is validated for the docking studies. Binding sites and ligand poses were correctly identified, as shown in Figure 1b. The redocked native ligand showed similar binding positions and orientations within the binding site and were similar to the co-crystal structures with root mean squares less than 2A (0.7864). The superimposition of the redocked native ligands with its co-crystal structure was also correctly reproduced (Figure 2) within the binding domain of the receptor (see Figure 1b).

#### 3.3.2. Preparation of Protein

The ligand-binding domain of the hERα protein was retrieved from the RCSB protein data bank with the PDB id 2IOG (see X-ray structure in Figure 1a). The resolution, species and bound structure were taken into consideration before choosing the target protein from the database. Protein preparation was processed with the help of the protein preparation wizard from the workflow option of the Schrodinger suite. The force field applied for the preparation of the protein was the optimized potentials for liquid simulations-2005. The water molecules, heteroatoms residues, were deleted while the chain was retained along with H-bond. Hydrogen atoms were added and such bonding network was optimized. Side chains and loops with missing atoms were also built. The complex obtained was finally minimized after the energy gradient converged below 0.05 kcal/mol using the OPLS_2005 force field with Polack-Ribiere Conjugate Gradient (PRCG) algorithm [41]. The receptor-grid was generated with the help of the module glide. Grid generation represents the physical properties like volume of the receptor (specifically the active site) that is needed for carrying out the ligand-docking. The grid boxes were generated by selecting the co-crystallized ligands in the ligand-binding domain and replacing it with the studied ligands during the docking process.

#### 3.3.3. Molecular Docking Studies

Good binders to the target receptor were investigated intensively using Glide extra precision (XP) docking for clear and accurate details along with epik state penalties. The selected entries for the ligands to be docked were being selected as well as the output file of the generated grid. The docking of each potential drug molecule along with the grid generated conformational changes with respect to the active site of the protein, estrogen receptor (PDB ID-2IOG). Following the docking studies, the glide scores or docking scores were displayed and the ligand amongst the phytomolecules, with the least glide score was considered to have the best docked pose or best glide score. The glide scoring system has been well established and has been deemed very accurate in comparison to other docking tools [28]. Glide score approximates the ligand binding free energy.

### 3.4. Prime Energy Analysis

For the post assessment of docked structures, the molecular mechanics energies combined with the Poisson–Boltzmann or generalized Born and surface area continuum solvation (MM/PBSA and MM/GBSA) methods were used [35]. These are popular approaches to estimate the free energy of the binding of small molecules to biological macromolecules [42]. The calculation uses the OPLS_2005 all-atom force field for protein residues as well as for ligands and cofactors. The input structures for these calculations were taken from a Pose Viewer file Glide output.

The following descriptors were generated by the Prime MM-GBSA approach:
MM-GBSA_∆G_bindLigand binding energy, $\Delta G_{bind}$MM-GBSA_E_complexEnergy of the complex, $G_{complex}$MM-GBSA_E_proteinEnergy of the receptor without the ligand, $G_{protein}$MM-GBSA_E_ligandEnergy of the unbound ligand, $G_{ligand}$

The total free energy of binding was then expressed as:

(1) ΔGbind= Gcomplex– (Gprotein+Gligand)

The other parameters were:
Prime Coulomb energy of the complex$\Delta G_{bind}$ coulombPrime Van der Waals energy of the complex$\Delta G_{bind}$ vdWPrime Hydrogen Bond of the Complex$\Delta G_{bind}$ Hbond

The MM-GBSA scoring along with the experimental binding affinity data of the binding site for studied molecules on 2IOG is presented in Table 3.

### 3.5. Preparation of Drugs

Artonin E used in these studies was isolated from the stem bark of *Artocarpus elasticus* [43], while Tamoxifen, the reference standard was purchase from Sigma Aldrich, St. Louis, MO, USA. A stock solution of 100 mM was prepared in DMSO and the final DMSO concentration was 0.01%.

### 3.6. Cell Culture

The MCF-7 cancer cell line was purchased from the American Type Culture Collection (ATCC, Rockville, MD, USA) and was maintained in RPMI media supplemented with 10% heat-inactivated FBS, 100 U/mL penicillin and 100 µg/mL streptomycin (Sigma). Cells were grown in 25 cm^2^ tissue culture flasks in a humidified atmosphere containing 5% CO_2_ at 37 °C. Once the cells reach 80% confluency, 1 mL of trypsin-EDTA solution was added to the flask to detach the monolayer cells. Approximately 0.5 × 10^6^–1 × 10^6^ cells were routinely sub-cultured and maintained appropriately.

### 3.7. Growth Inhibitory Assay

The growth inhibitory potential of Tamoxifen, a standard estrogen receptor modulator and Artonin E, the best glide scorer among the natural compounds from *Artocarpus* species was assessed using a cell-based proliferation assay in estrogen positive MCF-7 breast cancer cell line. Briefly, exponentially growing MCF-7 cells was seeded in 96-well micro-plates at a density of 0.5 × 10^4^ cells/well. The Cells were allowed to adhere overnight before being challenged with the compounds. The cells were incubated at various time points with different compound concentrations ranging from 1.56–100 µM. After each treatment time point, 20 µL of MTT (Sigma, St Louis, MO, USA) stock solution (5 mg/mL) was added to each well and incubated for 4 h to allow metabolization of the MTT by cellular mitochondrial dehydrogenases. One hundred microliters of DMSO was thereafter added to each well to solubilize the formazon crystals formed. The absorbance of the converted dye was measured colorimetrically at 570 nm and the assay was carried out in three independent experiments. From the obtained absorbance readings at respective intervals of each tested agent at each concentration, a nonlinear regression was performed using the GraphPad Prism software and the concentration of each agent that evoked a 50% growth inhibition of the estrogen positive breast cancer cells was determined along with their 95% confidence interval (Table 5). A dose–response curve was fitted for each of the compounds with the *X*-axis being the logarithm of the concentrations used and the *Y*-axis showing cell viability response calculated using the following formula:

(2) $$\%\text{ of cell viability}=\;\frac{A_{T}}{A_{C}}\;\times 100$$

where *A_T_* is the absorbance reading of treated samples at each time point, and *A_C_* is the absorbance of control samples treated with 0.01% of DMSO equivalent to the amount of DMSO used as a vehicle in the compound-treated wells.

## 4. Conclusions

Targeting the human estrogen receptor α, a strategy adopted in this study, is a valid approach in discovering new molecules which can halt undue proliferation in estrogen positive breast cancer. The occupation of the binding pocket of hERα by ER targeting small molecules can prevent estrogen from binding to such active site and thus abrogates estrogenic downstream signaling which precedes undue proliferation, as implicated in estrogen receptor positive breast cancer.

The present study used nine ligands from *Artocarpus* species to study their binding affinities to the ligand-binding domain of the human estrogen receptor α (PDB ID 2IOG) using the Glide module of Schrodinger software after ascertaining their drug-likeness with Qikprop. Artonin E, Artonin U and Cycloartocarpesin were the best amongst the phytomolecules based on their docking scores as well as the Prime MM-GBSA free energy of binding. The in silico study revealed that the studied phytomolecules are potent modulators of estrogen receptor positive breast cancer. The best glide scorer amongst the phytomolecules, Artonin E, was investigated for its time interval growth inhibitory effect on an ER positive breast cancer cell line, MCF-7 along with Tamoxifen, a standard estrogen receptor modulator. In vitro growth inhibition is an essential prerequisite of anticancer drug development, owing to the unique hallmark of cancer cells to resisting cell death and proliferating uncontrollably. Artonin E, with a glide score of 12.72 kcal, showed a better in vitro growth inhibitory effect in submicromolar range as compared to Tamoxifen (glide score of 13.93 kcal), a standard estrogen receptor modulator currently facing much reported resistance. Conclusively, in silico molecular studies have proven very useful in predicting the pharmacokinetic profiles and the binding affinities of suspected new drug candidates before a detailed preclinical and clinical evaluation. This study has revealed potent estrogen receptor modulators with good predicted pharmacokinetic profiles that should be further investigated for their in vitro as well as in vivo activity towards estrogen receptor positive breast cancer. We recommend that the enlisted analogs of Artonin E be screened for their potential anti-breast cancer effects as predicted by our in silico data.

## Figures and Tables

**Table 1:** Prediction of drug-likeness and pharmacokinetic profile of studied molecules.

Ligands	*M*_W_	SASA	Donor HB	Accepted HB	QPlogPo/w	QPlogS	QPPCaco	QPlogBB	# Metab	% Human-Oral Absorption	Lipinskis Rule of Five
**Artonin E**	436.5	734.1	3	5.3	3.9	−6.4	124.6	−1.9	7	88	0
**Artobiloxanthone**	434.4	689.9	3	5.3	3.6	−5.8	156.3	−1.6	7	87	0
**Cycloartocarpesin**	352.3	608.4	2	4.5	2.9	−5.1	193.3	−1.4	3	85	0
**Artoelastin**	488.6	821.3	2	4.5	6.2	−8.1	704.4	−1.2	12	100	1
**Artonin Y**	354.4	580.3	3	4.5	2.4	−4.0	75.4	−1.8	7	75	0
**Artonin U**	352.4	618.9	1	3.8	3.9	−5.4	380.3	−1.2	6	96	0
**Artonin L**	396.4	635.0	1	5.3	3.5	−5.3	461.1	−1.0	6	95	0
**Artonin T**	450.5	718.9	2	5.3	4.5	−6.3	451.0	−1.2	9	100	0
**Artonin S**	452.5	728.0	2	5.3	4.6	−6.4	333.7	−1.4	8	100	0
**Tamoxifen**	371.5	730.6	0	2.6	6.6	−5.9	2203.2	0.4	3	100	1
**Native Ligand**	547.9	1021.8	4	10.1	4.3	−4.9	20.7	−0.6	4	62.9	1

Range for 95% known drugs: Molecular weight (*M*_W_) = 130.0–725.0; Total solvent accessible surface area (SASA) = 300.0–1000.0; Donor HB = 0.0–6.0; Accept HB = 2.0–20.0; Predicted octanol/water partition coefficient (QPlogPo/w) = −2.0–6.5; Predicted aqueous solubility (QPlogS) = −6.5–0.5; Predicted apparent Caco-2 cell permeability (QPPCaco) ≤25 poor, >500 great; Predicted brain/blood partition coefficient (QPlogBB) = −3.0–1.2; Number of likely metablioc reactions (# metab) = 1–8; % Human oral absorption ≥80% →High, <25% →Poor; Number of violations of Lipinskis Rule of Five; mol MW < 500, QPlogPo/w < 5, donor HB ≤ 5, accpt HB ≤ 10. Compounds that satisfy these rules are considered drug-like.

**Table 2:** Glide scores of studied ligands with their electrostatic interactions (kcal/mol) with critical amino acid at the ligand-binding domain of hERα.

Ligands	Glide Score	THR 347		ASP 351		GLU 353		ARG 394	
		VDW	Coloumb	VDW	Coloumb	VDW	Coloumb	VDW	Coloumb
**Native ligand**	−16.81	−2.46	−3.23	−1.99	−50.43	0.63	−23.30	−0.24	7.52
**Tamoxifen**	−13.93	−2.35	−2.36	−1.47	−40.68	−0.85	−17.79	−0.26	13.57
**Artonin E**	−12.72	−2.35	−1.76	−1.03	−12.11	−1.42	−2.87	−0.60	1.21
**Cycloartocarpesin**	−11.72	−0.41	−0.23	−0.10	−1.09	1.19	−13.83	0.15	−1.67
**Artonin U**	−11.03	−3.21	0.27	−0.74	−1.51	−0.11	−11.60	−0.35	−1.43
**Artoelastin**	−10.90	−3.35	−0.11	−1.16	−1.32	−1.05	−10.05	−0.45	−1.58
**Artonin L**	−10.70	−1.11	0.36	−0.15	−0.34	−0.24	−1.45	−0.06	0.79
**Artobiloxanthone**	−10.50	−1.21	−1.26	0.69	−1.20	−0.22	2.33	−0.04	−1.63
**Artonin Y**	−10.50	−2.89	−0.77	−0.57	−1.93	−1.50	−1.21	−0.39	0.66
**Artonin T**	−9.10	−1.47	−0.26	−1.19	−1.45	−0.79	−1.17	−0.00	−0.01
**Artonin S**	−9.10	−2.80	−0.61	−2.99	1.04	−0.37	−3.18	−0.09	2.49

THR—Threonine, ASP—Aspartic acid, GLU—Glutamic acid, ARG—Arginine.

**Table 3:** Output properties from a Prime MM-GBSA calculation.

IUPAC Names	$\Delta G_{bind}$ (kcal/mol)	$\Delta G_{bind}$ Coulomb	$\Delta G_{bind}$ vdW	Prime MMGBSA Complex Energy	Prime MMGBSA Ligand Energy	Prime MMGBSA Receptor Energy	$\Delta G_{bind}$H Bond
Artonin E 5-hydroxy-8,8-dimethyl-3-(3-methylbut-2-enyl)-2-(2,4,5-trihydroxyphenyl)pyrano[2,3-*h*]chromen-4-one	−47.68	−22.59	−33.53	−9989.98	−119.08	−9823.22	−1.76
Cycloartocarpesin 8-(2,4-dihydroxyphenyl)-5-hydroxy-2,2-dimethylpyrano[3,2-*g*]chromen-6-one	−51.28	−18.48	−38.52	−10,022.60	−148.09	−9823.22	−2.13
Artonin U 5-Hydroxy-2-(4-hydroxyphenyl)-7-methoxy-8-(3-methyl-2-buten-1-yl)-4*H*-chromen-4-one	−60.35	−16.22	−52.39	−9987.84	−104.26	−9823.22	−1.99
Artoelastin 3,8,10-trihydroxy-9,11-bis(3-methylbut-2-enyl)-6-(2-methylprop-1-enyl)-6*H*-chromeno[4,3-*b*]chromen-7-one	−35.29	−13.48	−30.50	−10,017.30	−158.81	−9823.22	−1.65
Artonin L 3,8-Dihydroxy-1,10-dimethoxy-5,5-dimethyl-5a,6-dihydro-5*H*,7*H*-[1]benzofuro[3,4-*bc*]xanthen-7-one	−32.69	−5.91	−26.01	−9949.88	−93.968	−9823.22	−0.05
Artobiloxanthone 6,10,11,13-Tetrahydroxy-9-isopropenyl-3,3-dimethyl-8,9-dihydro-3*H*,7*H*-benzo[c]pyrano[3,2-*h*]xanthen-7-one	−11.32	−5.45	−16.28	−9930.81	−96.27	−9823.22	−0.15
Artonin Y 2-(2,4-Dihydroxyphenyl)-5,7-dihydroxy-8-(3-methyl-2-buten-1-yl)-4*H*-chromen-4-one	−44.70	−9.09	−47.89	−10,023.60	−155.67	−9823.22	−1.20
Artonin T 1,3,8-Trihydroxy-10-methoxy-5,5-dimethyl-2-(3-methyl-2-buten-1-yl)-5a,6-dihydro-5*H*,7*H*-[1]benzofuro[3,4-*bc*]xanthen-7-one	−27.36	−3.95	−46.21	−9953.46	−102.87	−9823.22	−0.24
ARTONIN S 3,9-dihydroxy-6-(2-hydroxypropan-2-yl)-11-methoxy-10-(3-methylbut-2-enyl)-6,7-dihydrochromeno[3,2-*d*][1]benzoxepin-8-one	−25.70	3.67	−28.01	−9957.42	−108.50	−9823.22	−0.33

See structure of ligands in Figure 3a–i.

**Table 4:** Hydrogen bonding interactions between studied ligands and the critical amino acids at the ligand-binding domain of hERα.

Ligands	No of Bonds	Residues	Distance
Artonin E	4	THR 347, ASP 351, LYS 531, CYS 530	2.22, 1.84, 3.04, 3.22
Cycloartocarpesin	3	ARG 394, GLU 353, LEU 346	1.94, 3.18, 2.70
Artonin U	3	GLU 353, ARG 394 GLY 521	1.81, 3.41, 3.44
Artoelastin	3	GLU 353, ARG 394, GLY 521	1.94, 2.31, 3.41
Artonin L	2	CYS 530, MET 528,	3.37, 2.08
Artobiloxanthone	2	LEU 525, MET 343	3.37, 3.29
Artonin Y	2	LEU387, ARG394	2.02, 2.25
Artonin T	1	ARG 394	3.50
Artonin S	1	THR 347	2.23
Native ligand	3	GLU 353, LYS 351,ARG 394	1.80, 2.08, 2.11
Tamoxifen	2	LYS 351, CYS 530	1.92, 3.51

THR—Threonine; ASP—Aspartic acid; LYS—Lysine; CYS—Cysteine; ARG—Arginine; GLU—Glutamic acid; LEU—Leucine; MET—Methionine.

**Table 5:** IC_50_ Values of Artonin E and Tamoxifen on MCF 7 at Different time interval.

Compounds	24 h		48 h		72 h	
	IC_50_ (µM)	95% Confidence Interval	IC_50_ (µM)	95% Confidence Interval	IC_50_ (µM)	95% Confidence Interval
**Artonin E**	6.9	5.5–8.6	5.1	4.5–5.8	3.8	3.4–4.1
**Tamoxifen**	24.1	22.6–25.7	20.6	18.6–22.9	18.9	17.5–20.4

## Supplementary Materials

The following are available online at http://www.mdpi.com/1420-3049/21/7/839/s1. In silico physicochemical data, prime energy analysis and in vitro raw data as well as the result analysis and graphs.
